# Individualized treat-and-extend regime for optimization of real-world vision outcome and improved patients’ persistence

**DOI:** 10.1186/s12886-020-01397-x

**Published:** 2020-03-30

**Authors:** Ingo Volkmann, Katharina Knoll, Mareile Wiezorrek, Oliver Greb, Carsten Framme

**Affiliations:** Hannover Medical School, University Eye Hospital, Carl-Neuberg-Straße 1, 30625 Hannover, Germany

**Keywords:** Anti-VEGF, Macular edema, Treatment strategies, PRN, TE, Intravitreal injection

## Abstract

**Background:**

Intravitreal injections are a mandatory treatment for macular edema due to nAMD, DME and RVO. These chronic diseases usually need chronic treatment using intravitreal injections with anti-VEGF agents. Thus, many trials were performed to define the best treatment interval using pro re nata regimes (PRN), fixed regimes or treat-and-extend regimes (TE). However, real-world studies reveal a high rate of losing patients within a 2-year interval of treatment observation causing worse results. In this study we analyzed retrospectively 2 years of real-world experience with an individualized treat-and-extend injection scheme.

**Methods:**

Since 2015 our treatment scheme for intravitreal injections has been switched from PRN to TE. Out of 102 patients 59 completed a follow up time of 2 years. Every patient received visual acuity testing, SD-OCT and slit lamp examination prior to every injection. At each visit an injection was performed and the treatment interval was adjusted mainly on SD-OCT based morphologic changes by increasing or reducing in 2-week steps. Individual changes of the treatment protocol by face-to-face communication between physician and patient were possible.

**Results:**

After 1 year of treatment visual acuity gain in nAMD was 7.4 ± 2.2 ETDRS letters (*n* = 34; injection frequency: 7.4 ± 0.4) respectively 6.1 ± 4.7 in DME (*n* = 9; injection frequency: 8.4 ± 1.1) and 9.7 ± 4.5 in RVO (*n* = 16; injection frequency: 7.6 ± 0.5). After 2 years of treatment results were as following: nAMD: visual acuity gain 6.9 ± 2.1 (injection frequency: 12.6 ± 0.7); DME: 11.1 ± 5.1 (injection frequency: 14.0 ± 1.0); RVO: 7.5 ± 5.0 (injection frequency: 11.2 ± 0.9). Planned treatment exit after 2 year was achieved in 29.4% of patients in nAMD (0% after 1 year); 0% in DME (0% after 1 year); and 31.3% in RVO (0% after 1 year). Patients’ persistence was 94.1% during the follow-up.

**Conclusion:**

Using a consequent and individualized TE regime in daily practice may lead to a high patients’ persistence and visual acuity gains nearly comparable to those of large prospective clinical trials. Crucial factors are face-to-face communication with the patient as well as a stringent management regime. At this time TE may be the only instrument for proactive therapy which should therefore be regarded as a first-line tool in daily practice.

## Background

Neovascular age related macular disease (nAMD), diabetic macular edema (DME) and retinal vein occlusion (RVO) are common causes for macular edema and related vision loss [1]. Intravitreal antibodies against vascular endothelial growth factor proofed to reduce macular edema and to prevent visual loss [2–4]. Since the start in treating nAMD with VEGF-inhibitors on an on-label base in the year 2006 using Ranibizumab many studies have been performed to evaluate the best injection frequency and also optimal exit strategies. Since Ranibizumab therapy was introduced in Europe as a pro re nata regime (PRN) starting with three injections at 4-week intervals, it has taken several years to learn, that in real world patients were not optimally treated. Physicians needed to understand that e.g. nAMD is a chronic condition, which needs to be treated on a “chronic” and continuous base. However, due to high costs of the new drug and a sophisticated chronic patients management, the required injection frequencies to stop fluid accumulation and the very good visual acuity results of the large prospective clinical trials using monthly injections [2, 3, 5] were not achieved. Recent real world data reveals injection frequencies of only 4.3 during the first year in Germany for treating nAMD [6, 7] instead of 12 injections as given in ANCHOR or MARINA [2, 3]. Other European countries revealed same problems of undertreatment leading to a complete loss of the visual gain only 2 years after treatment start [6]. Regarding to AURA Great Britain achieved highest visual gain (+ 4.1 ETDRS letters) after 2 years using 9 injections in contrast to Germany showing − 0.8 ETDRS letters after only 5.6 injections [7]. Thus, injection frequency seems to be a crucial factor for achieving high and constant visual gains. The real world observational German PERSEUS study using Aflibercept showed, that not only injection frequency but also adequate continuous treatment is important to achieve stable visual gains [8]. Especially an observed larger time gap between the third and the fourth injection (“upload break”) leads to visual losses, which could not be improved later on [8]. Also, other real-world observational studies as AURA [7] and WAVE [9] showed same insufficient results in treating nAMD on a continuous base.

In nAMD it is important to avoid disease activity optimally injecting Anti-VEGF before new fluid appears, which always leads to irreversible structural damage. Since there are no other biomarkers for disease activity than SD-OCT detected macular fluid, PRN’s problem is “reactivity”. Treatment is always given, if fluid already appeared. In a strict PRN protocol with 4-week control intervals and higher injection frequencies (e.g. “always 3 injections in case of retinal fluid”) high and constant visual acuity results can be achieved also in real world [10]. Here it was shown, that using an injection frequency of about 7–8 injection during the first year, visual acuity gain comparable with prospective clinical trials using monthly injections, can be reached. However, such strict regimens are obviously difficult to implement in clinical setting; because it needs many control visits, many decisions and as explained always new fluid activity.

Based on the obvious real world undertreatment new drug labels as for Aflibercept allowing a first-time fixed treatment every eigth weeks over 1 year seemed to be a good step to enhance treatment results; however, it did not lead to an optimal injection frequency in Germany, although a mean of about 5.8 injections were given during the first year [8]. Interestingly, in that study only 25% of all patients were treated fixed regarding to the label leading to better results than those who were not [8]. Moreover, there was a high drop-out rate of patients of about 65% after 2 years (data not published). This is consistent with other real-world observations showing about 50% drop-out after only 1 year and about 70% after 2 years [6]. Thus, patients’ persistence is a very crucial factor in treating macular edema by intravitreal injections meaning high efforts and costs.

A fixed regimen bears a high risk of under- and overtreatment, because injections were given on a fixed base independent from the “morphologic need” as usually determined by SD-OCT. However; huge heterogeneity is observed for the time span of dry retina after one injection in all three macular diseases (nAMD, DME, RVO). Mantel et al. could show, that patients who were able to obtain and hold dry macular conditions for e.g. eigth weeks after injections, also later on usually were able to achieve at least the same” dry periods” [11]. Thus, a TE management seems to be a feasible instrument to inject Anti-VEGF in a proactive manner before fluid reactivation appears by individually testing the time span of dryness between two injections. Based on SD-OCT evaluated “dryness” the next injection interval is extended for e.g. two weeks as well as reduced by e.g. one or 2 weeks in case of reactivated fluid.

Carefully explaining the patient the need for such a strict but individualized treatment system for chronic macular diseases may lead to better understanding and better adherence. SD-OCT is usually performed directly before the treatment and the next interval is determined. This management gives high transparency to the patient and he knows, that at every visit an injection will be performed. Given that the patients’ adherence to treatment is well, one problem is the treatment stop. There is no consensual agreement about definite stop criteria. However, many specialists think that after a “dry” period of about 16 weeks a treatment stop should be considered [12].

The purpose of our study was to evaluate, whether an individualized TE regimen can be implemented in a real-world clinical setting of a tertiary referral center and to evaluate visual acuity results based on such new treatment strategies as well as determining patients’ persistence.

## Methods

In 2015 treatment strategy was switched from PRN to TE for all three main macular diseases as nAMD, DME and RVO. From actually 102 patients, who were treated on an individual base by mainly one surgeon (CF) in an “individual TE system”, we evaluated retrospectively 59 patients (nAMD: *n* = 34; DME: *n* = 9; RVO: *n* = 16) with a complete 2-year follow up. For patients with shorter treatments we still included these for calculation of total patient loss and treatment stop. Included were patients over 50 years of age with choroidal neovascularization due to nAMD who were treatment-naïve or have had no injection therapy at least 6 months prior to inclusion. All DME and RVO patients were treatment-naïve and presented with subretinal or intraretinal macular fluid based on SD-OCT examination. With regard to Giannakaki-Zimmermann et al. ([12]) we established a strict TE management system using injection interval extensions mainly beginning from the first injection. In detail beginning with the first injection, every patient receives a macular SD-OCT using a volume scan of 49 scans, visual acuity testing and slit lamp examination of the anterior segment prior to every injection. No mydriatic funduscopy is performed. Based on SD-OCT the injection period (minimum: four weeks) was selected as following: with the baseline OCT first injection is given and patient is scheduled 4 weeks later for second injection. Then, in case of a dry-stage macula in SD-OCT, patient receives injection and the injection interval is extended by 2 weeks, which means third injection is scheduled after 6 weeks. The interval is extended usually on 2-week steps in cases of dry retina conditions and reduced individually by one to 2 weeks in cases of reactivated fluid. No upload or new “4-week interval” injection period is started after fluid reactivation. In cases of persistent macular fluid without complete resolution the injection interval is kept stable on 4 weeks. No intervals shorter than 4 weeks are indicated on a regular base. In cases of dry retina up to 14 weeks, the “last” injection is given at the time point “14 weeks” and the patient is scheduled for control visit at week 16 without planned injection (“EXIT strategy”). If the macula is dry at this time point, another control visit will be planned four to 6 weeks later on for possible reactivation control. Bilateral diseases were included in this study and treatments were planned for each eye individually.

We retrospectively analyzed the individualized TE injection scheme with focus on individualization by morphological parameters. Therefore, medical records and SD-OCT images of all patients with nAMD, DME and RVO, who were scheduled for intravitreal injection using Anti-VEGF (Ranibizumab, Aflibercept and Bevacizumab) by surgeon CF and who completed a 2-year follow-up, were retrospectively analyzed. In accordance with our local ethics committee, no ethical approval for retrospective analized data was needed (Ethics Committee, Hannover Medical School, Hannover, Germany). The study was performed in accordance with ICH-GCP guidelines. A written consent for collecting data for scientific reasons was obtained by each patient.

BCVA was measured using autorefraction methods (Topcon KR-800S) and Decimal visual acuity was recalculated to ETDRS letters. Tonometry was performed with Nidek NT 510 noncontact tonometer followed by Goldmann applanation in case of increased intraocular pressure. Slit lamp examination of the anterior segment to rule out possible inflammation was performed using Haag Streit BQ 900 LED. SD-OCT images were obtained using Spectralis OCT (Heidelberg Engineering, Heidelberg, Germany, Software 6.7.13). Qualitative evaluation was obtained by the surgeon immediately before injection by fluid detection using standard macular volume scans. For the volume scan of 20 × 20 grade 49 frames spaced 121 μm and each consisting of 512 A-scans were acquired. Usually injection interval was extended in case of no fluid detection (“zero fluid tolerance”). The patient was extensively educated about the proactive nature of the chronic macular disease and the preferred TE management system.

Intravitreal injections were performed in a class 3 operating room. Anesthesia was performed using Proparacaine eyedrops. For skin disinfection Povidone Iod 7.5% was used, the conjunctival disinfection was completed by 1:1 Povidone Iod dilution, each for a minimum application period of 30 s. Povidone Iod was rinsed with NaCl prior to injection. Sterile covers were used as well as a lid opener. Intravitreal injection was performed in 3.5 mm distance from the limbus. Postoperatively, hand motion testing was performed as well as antibiotic ointment application. No further bandage was applied. No postoperative control visit was scheduled but only the next injection visit. Each patient was clearly explained the definite symptoms of endophthalmitis and immediate control in clinics in case of the onset of such symptoms.

The primary endpoint of this study was visual gain explained by ETDRS letters after one and 2 years of treatment as well as the corresponding injection frequency independent of the specific anti-VEGF agent used. Since the drug impact is regarded as less important than the proactive management system in terms of achieving best visual gain [2, 3, 5, 13], no drug differentiation was considered in this study. Secondary endpoints consisted of percentage of planned EXITs from injection therapy as well as the overall patients’ persistence to the continuous treatment as advised by the surgeon.

Ended treatments due to worsening of atrophy, non-responding to intravitreal injections or other reasons were counted as a stop. In case of missed treatments the patients most often did a reschedulement by themselves. If the patient did not show up and did not made a new appointment we asked actively by telephone if the patient wanted to quit, could not come due to health issues or if he or she just forget to make a new appointment.

## Results

Out of 59 patients 34 were treated for nAMD, 9 patients for DME and 16 patients for RVO. Independently of these diseases the same TE management was mainly applied. The overall mean injection frequency during the first year was 7.6 injections, in the second year 4.8 injections were given. Thus, in summary 12.4 injections have been applied during the 2-year period.

Mean visual acuity at baseline for all diseases was 56.5 ± 2.4 ETDRS letters and changed to 64.2 ± 2.4 ETDRS letters after 2 years of treatment with a mean gain in 7.7 ± 1.9 ETDRS letters.

**Fig. 1:**
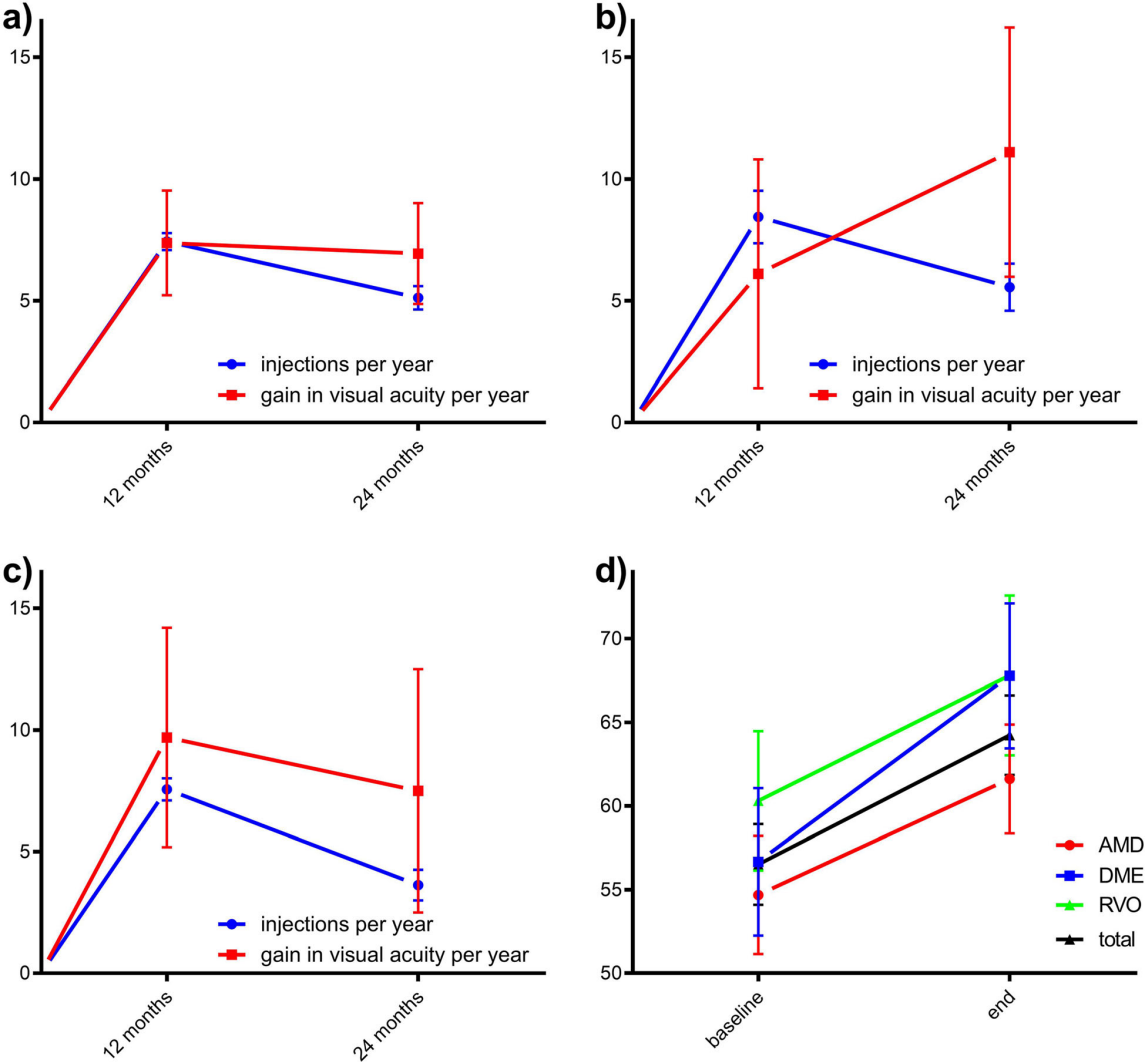
Injections per year and gain in visual acuity per year for (**a**) nAMD, **b** DME and **c** RVO and baseline as well as (**d**) end-ETDRS letters. [injections] = numbers, [visual acuity] = amount of EDTRS-letter-increase. Data are mean ± SEM. nAMD *n* = 34, DME *n* = 9, RVO *n* = 16

Regarding the specific diseases after 1 year of treatment visual acuity gain in nAMD was 7.4 ± 2.2 ETDRS letters (*n* = 34; injection frequency: 7.4 ± 0.4) respectively 6.1 ± 4.7 in DME (*n* = 9; injection frequency: 8.4 ± 1.1) and 9.7 ± 4.5 in RVO (*n* = 16; injection frequency: 7.6 ± 0.5). After 2 years of treatment results were as following: nAMD: visual acuity gain 6.9 ± 2.1 (injection frequency: 12.6 ± 0.7); DME: 11.1 ± 5.1 (injection frequency: 14.0 ± 1.0); RVO: 7.5 ± 5.0 (injection frequency: 11.2 ± 0.9). All data including baseline and end ETDRS values are listed in Table 1. Average injections per year and gain in visual acuity is shown in Fig. 1 including baseline and end ETDRS values.

**Table 1:** Summary of injection frequency, gain in visual acuity and EXIT after one and 2 years for patients with neovascular age related macular disease (nAMD), diabetic macular edema (DME) and retinal vein occlusion (RVO). Data are mean ± SEM

		nAMD (*n* = 34)	DME (*n* = 9)	RVO (*n* = 16)
**Year 1**	injection frequency	7.4 ± 0.4	8.4 ± 1.1	7.6 ± 0.5
	gain in visual acuity [ETDRS]	7.4 ± 2.2	6.1 ± 4.7	9.7 ± 4.5
	EXIT	0%	0%	0%
**Year 2**	injection frequency	12.6 ± 0.7	14.0 ± 1.0	11.2 ± 0.9
	gain in visual acuity [ETDRS]	6.9 ± 2.1	11.1 ± 5.1	7.5 ± 5.0
	EXIT	29.4%	0%	31.3%
**ETDRS**	Baseline	54.7 ± 3.5	56.7 ± 4.4	60.3 ± 4.2
	End	61.6 ± 3.3	67.8 ± 4.3	67.8 ± 4.8

**Fig. 2:**
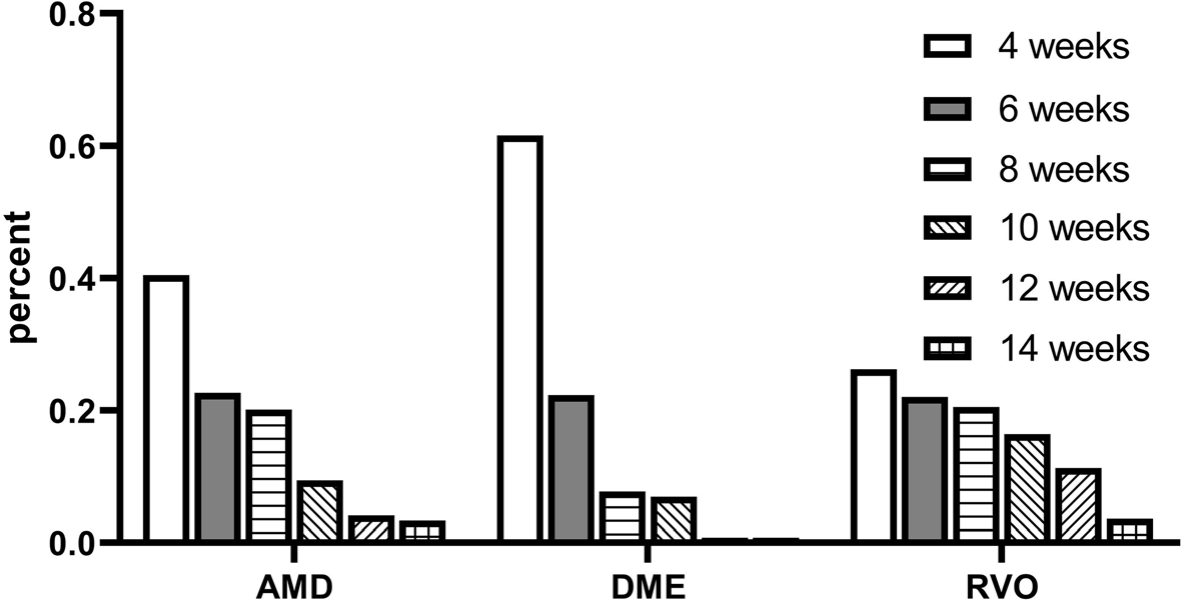
Injection interval per disease in percent of all injections for that disease. [injections] = numbers. Data are total injection numbers. nAMD *n* = 34, DME *n* = 9, RVO *n* = 16

In total, 732 injections were performed within 2 years in 59 patients. Analyzing injection frequency, 40.4% of all injections were performed every 4 weeks, whereas 22.8% of all injections were performed after 6-week intervals, 18.3% of all injections were performed after 8-week intervals, 10.7% of all injections were performed after 10-week intervals, and finally 5.7% of all injections were performed after 12-week intervals and 3.0% of all injections were performed after 14 weeks (Fig. 2).

Notably, 20.6% of all patients in nAMD (*n* = 7) needed injections every four to 6 weeks within 2 years which means that they never reached a stable, dry stage which would allow an extension of treatment intervals. Moreover, 55.6% of all patients in DME (*n* = 5) needed injections every four to 6 weeks in 2 years and only one patient in RVO, indicating best therapy responses in RVO but worst responses in DME.

**Fig. 3:**
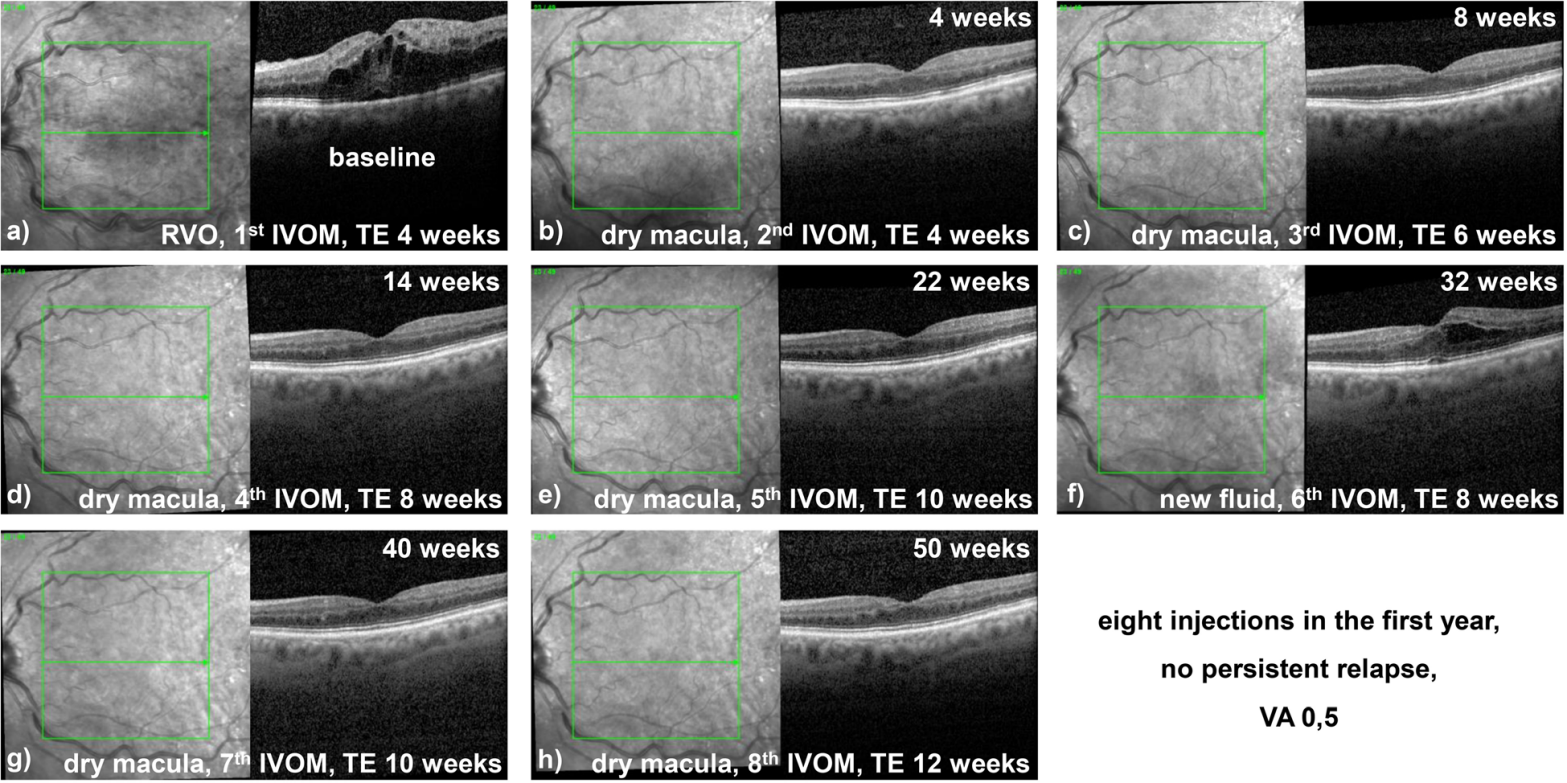
TE injection scheme in RVO (baseline visual acuity 0.1): **a**) baseline and 1st injection. **b**) dry macula after first injection, extend possible but postponed in shared decision making with the patient. **c**) dry macula, now extension of intervals to enable a personalized regime. **d**) dry macula, further extension. **e**) dry macula, further extension. **f**) new fluid after extension to 10-week interval, 6th injection and fallback to last stable interval (8 weeks). **g**) dry macula after 8 weeks, new attempt for 10-week interval extension. **h**) dry macula 10 weeks after last injection, attempt to extend to 12 weeks. In total, eight injections were needed in the first year and a stable visual acuity gain to 0.5 was maintained

This implies that 79.4% of all patients in nAMD (*n* = 27) were able to reach longer injection intervals by eigth weeks showing enhanced stable dry stage macula. Accordingly, 44.4% of all patients in DME (*n* = 4) were able to achieve longer intervals on at least an 8-week base and even 93.8% of all patients in RVO (*n* = 15). Furthermore, 47.1% of all patients in nAMD (*n* = 16) were able to reach planned intervals longer than 10 weeks as well as 11.1% of all patients in DME (*n* = 1) and 68.8% of all patients in RVO (*n* = 11). A typical individualized TE regime in a case of RVO is displayed in Fig. 3.

Calculating the minimum time needed for an EXIT within our TE system, extension from 4 weeks successively to 16 weeks by increasements of 2 weeks between injections needs at least 54 weeks of treatment (see discussion). Considering this minimum time to EXIT, no patient in nAMD, DME or RVP reached an EXIT within 1 year. After 2 years, an EXIT for 15 patients out of 59 patients (37.3%) could be achieved. In detail, 29.4% of nAMD patients and 31.3% of RVO patients reached an EXIT within 2 years whereas all DME patients failed to reach an EXIT within 2 years. As all three antiVEGF drugs are used for TE we did not separate our data for the different drugs individually.

When considering all eyes treated in our cohort of TE patients, 18 out of 102 treatments were stopped during the course of intravitreal injections. For nAMD patients worsening of areolar atrophy or central scarring with led to treatment stop (*n* = 11). For DME patients disease progression with immobility or hospitalization stopped injection therapy (*n* = 5) and for RVO patients missing response of intravitreal fluid on intravitreal injections (*n* = 2) lead to a stop. From 102 eyes only 6 patients were lost due to the patients’ wish to exit the treatment, either by not showing up again or by clearly stating that they wish to exit the treatment for different reasons. Thus, patients’ persistence was 94.1% during the whole follow up.

## Discussion

TE management combines a fixed continuous treatment regimen and a PRN system in a proactive manner by variation of injection intervals avoiding the disadvantages of both regimes (fixed: non-individualized over- and undertreatment; PRN: recurrent disease activation, fluid accumulation and irreversible tissue damage). Compared to a regular PRN upload with 3 monthly injections TE will lead to an extension of this period of only 2 weeks, if complete fluid resolution is observed 4 weeks after first injection. Then the time interval is extended to 6 weeks between second and third injection. If no fluid resolution was achieved, patients would be treated maximally 12 times within the first year on a monthly base; if no fluid was observed within the first year of TE course 6–7 injections would be applied (Baseline 0: 1st injection is given; add 4 weeks to 4 weeks leads to 2nd injection; add 6 weeks to 10 weeks leads to 3rd injection; add 8 weeks to 18 weeks leads to 4th injection; add 10 weeks to 28 weeks leads to 5th injection; add 12 weeks to 40 weeks leads to 6th injection; and finally add 14 weeks to 54 weeks leads to 7th injection with a potential EXIT 16 weeks later. This visit might be already planned without injection. Thus, in face of having no different biomarkers for disease activation, TE possibly avoids under- and overtreatment on an individual but continuous injection frequency manner using as many injections as necessary.

The aim of the real-world treatment should be obtaining a stable visual acuity gain ideally comparable to those of referenced randomized prospective clinical trials [2, 3, 5, 14–18]. For nAMD mean visual acuity gains were 6.6 ETDRS letters over 2 years of monthly treatment using Ranibizumab [19] respectively 10.7 letters for completely classic CNV [2]. Given, there are about 15% of classic CNV, a letter increase of about 7.2 letters should be obtained in real-world settings treating all subgroups of nAMD. Study results using aflibercept in a fixed regime of monthly or bimonthly injections revealed same amount of visual gains [5, 20, 21]. Switching to a PRN regimen usually leads to worse vision results [22–24]. Regarding DME fixed monthly injections of Ranibizumab 0.3 mg lead to visual gains of about 12 ETDRS letters after 2 years [14]. PRN regimes, as performed in Europe with 0.5 mg dosages in randomized clinical trials, led to lower visual gains of about 7–8 ETDRS letters at an injection frequency of about 7 injections [25, 26]. Highest visual gains are usually observed in randomized clinical trials for Anti-VEGF treatment in RVO showing an ETDRS letter up to 21 letters, if predominantly fixed regimens are used [27–29].

Especially in nAMD but also in DME and RVO it is found that real world data revealed worse results than found in randomized trials predominantly because of low injection frequencies [6–9]. Moreover, a high loss of patients during treatment could be observed in such studies. It was our aim to evaluate, whether a comparable visual gain to randomized trials is possible to achieve also in a real-world setting, if a consequent TE regimen is performed. Since we learned that patients’ compliance to chronic therapy may significantly depend to enhanced medical elucidation, explanation and transparency, a proactive manner of “shared decision making” together with the patient was implemented to plan next therapeutic steps at each visit (“TE planning”).

In fact, it could be shown in our study, that after 1 year of treatment the overall visual gain was 7.8 ± 2.2 ETDRS letters, which is in accordance of study results of clinical trials as explained above. Moreover, visual gain was kept stable within the second year of treatment also comparable to clinical trials results [2, 3, 5, 14–18]. No significant visual loss during treatment as mainly seen in all real-world studies [6, 8, 9] could be observed. In detail, for nAMD an appropriate mean injection frequency of 7.4 led to an appropriate visual gain of 7.4 ETDRS letters. This result could be stabilized after 2 years with a high visual gain of 6.9 letters using 12.6 injections. For DME a comparably high first year injection frequency led to a mean of 6.1 letters visual gain, which seemed to be little lower than expected; however, it could be stabilized on an appropriate level of 11.1 letters visual gain after 2 years using 14 injections. For patients not being able to pursue monthly injections a switch to second line medications like intravitreal dexamethasone may be favorable [30].

Regarding RVO it could be learned from our results, that an early EXIT strategy leads to worse visual gains during the treatment course. Starting with a relatively high injection frequency of 7.6 after 1 year and a reasonable visual acuity increase of about 9.7 ETDRS letters, after 2 years the visual gain decreased to 7.5 letters and overall injection frequency during 2 years was only 11.2. Especially RVO patients often reveal SD-OCT based dry and stable macular conditions within the first injections. Then patients do not notice any visual problems and ask for a stop of intravitreal injection therapy. Facing these often-observed discrepancies between the need of continuous treatment and the somewhat “healthy” macular conditions, earlier EXIT strategies were discussed with the patient in the first year of TE management. As displayed in Tab. 1, highest planned EXIT rates were observed for RVO leading to a visual acuity gain of only 7.5 letters after 2 years, which is regarded as too low for RVO patients if compared to clinical trial results [27, 28, 31–33]. Also other studies suggest that a higher injection rate preserves a better visual outcome [34]. Thus, we changed our strategy to a more strict and rigid TE up to 14-week injection intervals even for RVO patients, who showed favorable visual gains in the early phase of treatment. Such rigid systems were adopted to nAMD on a regular base and also to DME. There were no EXITs during 2 years of treatment for DME patients indicating the chronical and mostly worsening course of the disease. This normally leads to a high number of injections as it could also be shown in our study.

Interestingly for nAMD revealing comparable good results as in clinical trials a planned EXIT could not be achieved after 1 year but 29.4% were achieved after 2 years. Herein, last injection was usually given at a 14-week dry interval after last injection. This moderate EXIT frequency after 2 years reveals the chronic nature of the disease with most patients needing more than 2 years and a larger amount of injections to reach a dry stage macula. In contrast to DME, disease activity of nAMD might be significantly reduced during continuous Anti-VEGF treatment leading to potentially inactive and dry retinal conditions. However; even after 16 weeks of “dryness” the risk of reactivity has to be regarded as high. Thus; after EXIT patients needed to be observed by SD-OCT on a regular and close-meshed base. Our management provides re-evaluation after EXIT at week 16 and then four weeks thereafter followed by another 6–8-week interval.

Compared to real-world studies our results reveal high visual gains at reasonable injection frequencies which moreover nearly reaches those results of clinical trials. In accordance with this successful TE strategy, the persistence quote of our patients was extremely high at about 94% after 2 years. We believe that it is particularly important to clearly discuss with the patient the chronic nature of the macular disease and the need for chronic therapy. Facing this importance of patients-physicians interaction we provide “shared decision making” at each visit. This does not mean to skip an injection but to carefully find together the schedule for the next injection visit, which can be held stable due to specific circumstances or can be increased by one or 2 weeks in dependence of other circumstances. However; it is essential to avoid a stop of the continuity of the treatment within the TE period up to 16 weeks. Larger time gaps of injection intervals regularly lead to new fluid accumulation, tissue damage and visual loss. The German real world study PERSEUS could impressively show that patients, who did not receive the 4th injection after the regular upload in nAMD at the scheduled interval but significantly later, lost visual acuity which could not be regained within the first year of treatment [8].

It is absolutely necessary to give impulses for a physician’s rethinking in terms of an appropriate therapeutic approach gone from “upload – wait and see” towards “continuous treatment”. Therefore, it needs to be learned that a “dry retina” does not mean “healthy retina” and thus injection therapy needs absolutely to be continued even in “dry retina”. Latter one only means a temporary more or less inactive disease condition but not a healthy one. General real-world data show expressively by revealing such low injection frequencies that physicians in daily practice still resist to inject in dry retinal conditions. This may lead to worse results and consequently a high loss of patients who may think that therapy does not lead to proper results.

In summary we could show that real word injection therapy for macular diseases as nAMD, DME and RVO may lead to considerable fine results somewhat comparable to randomized clinical trials if a strict but individualized TE system is applied. Finding the appropriate interval can be realized easily by SD-OCT and such a pro-active treatment scheme [11]. This approach can prevent an over-treatment and simplify the treatment by extending intervals whereby the patient notices an improvement and stabilization of visual acuity in between injections (positive feedback).

TE shows several advantages compared to PRN: the patient knows that at each visit an injection will be performed and it is much easier to schedule visits and guide patients in daily practice, because no uncertainties about the treatment exist. If patient and physician act together closely in terms of treatment continuity, best and stable visual gains as well as a high patients’ persistence can be obtained. Usually TE leads to a slightly higher injection frequency than PRN but to a considerably lower visit frequency, which is regarded as a positive aspect for chronic therapy from the patient’s point of view. For DME the RETAIN study could show, that there was a 40% reduction of monitoring visits in TE in contrast to PRN while achieving comparable visual gains over 2 years and 2 more injections in TE [31, 35]. For nAMD the TREND study revealed that a TE regimen leads to a comparable visual gain compared to a monthly injection therapy using ranibizumab after 1 year. For 6.6 letters visual acuity gain 8.7 injections were given. Interestingly over 60% of the TE patients achieved “dry intervals of at least eigth weeks and about 40% reached also 10 weeks [36]. In comparison to our results 7.4 injections for 7.4 letters visual acuity gain were given in the first year. After 2 years, 79.4% of TE patients reached injection intervals longer than eigth weeks and 47.1% of TE patients reached injection intervals longer than 10 weeks. The results of the randomized “TREND” clinical trial are in accordance to our results impressively showing that in fact chronic injection therapy can also be successful for patients on a long-term base in a real-world setting and appears to be safe as already evaluated by Giannakaki-Zimmermann et al. from the Berner Group in Switzerland [12]. They could show that switching from PRN to TE in 32 patients using aflibercept resulted in stable visual gains but a significant decrease of central retinal thickness under TE. Herein; injection frequency increased from 7.5 (PRN) to 10.25 (TE) after 1 year for each management system [12]. Reaching a real-world setting of PRN with 7.5 injections is an extraordinary positive exception with respect to the discussed real world studies as OCEAN and AURA [6, 7]. If so, the difference to TE seems to be small. However, as explained above clinical decision management, patients’ guidance and persistence as well as best visual acuity results are easier achieved using TE, which therefore should be used as the favorite system in clinical injection therapy.

## Conclusion

Using a consequent and individualized TE regime in daily practice may lead to a high patients’ persistence and visual acuity gains nearly comparable to those of large prospective clinical trials. Crucial factors are face-to-face communication with the patient explaining the need for continuous treatment as well as a stringent management regime. At this time TE may be the only instrument for proactive therapy avoiding under- or overtreatment, which should therefore be regarded as a first-line tool in daily practice.

## Data Availability

The datasets analysed during the current study are not publicly available because they are individual perioperative patients’ data but they are available from the corresponding author on reasonable request.

## Abbrevations

nAMD: Neovasular age related macular disease
DME: Diabetic macular edema
RVO: Retinal vein occlusion
PRN: Pro re nata
TE: Treat and Extend
SD-OCT: Spectral domain optical coherence tomography
